# Microbial diversity and soil physiochemical characteristic of higher altitude

**DOI:** 10.1371/journal.pone.0213844

**Published:** 2019-03-15

**Authors:** Saurabh Kumar, Deep Chandra Suyal, Amit Yadav, Yogesh Shouche, Reeta Goel

**Affiliations:** 1 Department of Microbiology, College of Basic Sciences and Humanities; Govind Ballabh Pant University of Agriculture and Technology, Pantnagar, Uttarakhand, India; 2 National Centre for Microbial Resource, National Centre for Cell Science, Sutarwadi, Pashan, Pune, Maharashtra, India; Babasaheb Bhimrao Ambedkar University, INDIA

## Abstract

Altitude is the major factor affecting both biodiversity and soil physiochemical properties of soil ecosystems. In order to understand the effect of altitude on soil physiochemical properties and bacterial diversity across the Himalayan cold desert, high altitude Gangotri soil ecosystem was studied and compared with the moderate altitude Kandakhal soil. Soil physiochemical analysis showed that altitude was positively correlated with soil pH, organic matter and total nitrogen content. However soil mineral nutrients and soil phosphorus were negatively correlated to the altitude. RT-PCR based analysis revealed the decreased bacterial and diazotrophic abundance at high altitude. Metagenomic study showed that *Proteobacteria*, *Acidobacteria* and *Actinobacteria* were dominant bacteria phyla at high altitude soil while *Bacteroidetes* and *Fermicute*s were found dominant at low altitude. High ratio of Gram-negative to Gram positive bacteria at Gangotri suggests the selective proliferation of Gram negative bacteria at high altitude with decrease in Gram positive bacteria. Moreover, *Alphaproteobacteria* was found more abundant at high altitude while the opposite was true for *Betaproteobacteria*. Abundance of *Cytophaga*, *Flavobacterium and Bacteroides* (CFB) were also found comparatively high at high altitude. Presence of many taxonomically unclassified sequences in Gangotri soil indicates the presence of novel bacterial diversity at high altitude. Further, isolation of bacteria through indigenously designed diffusion chamber revealed the existence of bacteria which has been documented in unculturable study of WIH (Western Indian Himalaya) but never been cultivated from WIH. Nevertheless, diverse functional free-living psychrotrophic diazotrophs were isolated only from the high altitude Gangotri soil. Molecular characterization revealed them as *Arthrobacter humicola*, *Brevibacillus invocatus*, *Pseudomonas mandelii* and *Pseudomonas helmanticensis*. Thus, this study documented the bacterial and psychrophilic diazotrophic diversity at high altitude and is an effort for exploration of low temperature bacteria in agricultural productivity with the target for sustainable hill agriculture.

## Introduction

Altitude is the major factor which has confounded effects on both biodiversity and soil physiochemical properties. High altitude ecosystems are generally characterized by low temperature, variable precipitation, decreased atmospheric pressure and soil nutrient stress which have major impact on biodiversity [1]. High altitude cold environments represent the majority of the biosphere on Earth and have been successfully colonized by cold adapted microorganisms that are able to thrive, better survive and even maintain metabolic activity at subzero temperatures [2].

Western Indian Himalaya (WIH) is complex ecosystem of the planet earth which is decorated with wide range of mountains, glaciers, hot springs, cold affected soils and cold deserts. The largest proportion of Himalayan soil habitats is comprised of high altitude areas with cold and nutrient deprived soil. Heavy snowfall and repeated freezing and thawing due to seasonal variation in climate create more adverse environment [3]. Such variations in microenvironment with high altitude change the ecosystem properties which is the major factor determining the diversity of microorganims in such extreme habitats.

Extreme climatic conditions and topography of Himalaya affects the soil physiochemical properties of cold deserts. However on the other hand, increasing pressure for food and fodder on agriculture is forcing it to increase cultivation in high altitude soil ecosystem. Variable climate, precipitation, temperature, seasonal variation and snowfall alter the soil microenvironment regularly which ultimately leads to the poor soil properties. The physiochemical properties of high altitude soil were reported to vary drastically with altitudinal gradient [4]. The soil in these ecosystems is coarse textured, deserted and poor in nutrients [5]. Various soil fertility characteristics like, organic carbon, pH, total nitrogen, phosphorus and micronutrients has been found to show altitudinal variation [6]. In spite of these facts, very few studies highlighted the effect of altitudinal gradient on soil physiochemical properties of WIH, thus relation of the soil properties with altitude remain obscure. Therefore, there is immense need to study the dynamics of soil physiochemical properties across the altitudinal gradient of WIH.

Moreover, soil bacterial communities from high altitude are regarded as among the most complex and diverse assemblages of microorganisms [7]. Majority of the microorganisms in high altitude are psychrophiles and psychrotrophs which are equipped with well adaptation to thrive in high altitude associated stresses. Psychrophilic microorganisms are extensively sought for the production of cold active enzymes, cold adapted biofertilizers and metabolites which work best at low temperature [8]. WIH has been extensively studied through culturable and unculturable approaches which revealed it as a hotspot for the psychrophilic and psychrotrophic microorganims [9–11]. However, most of these studies were conducted at low to moderate altitude of WIH, leaving the high altitude mountains and glaciers soils least explored. Further, very little is known about the variation in microbial community structure of WIH in response to the change in altitude. Therefore, exploration of the microbial diversity at high altitude soil ecosystems and their comparison with microbial diversity from attitudinally different soil would provide the better insight on the effect of altitude on microbial community composition.

Gangotri, the second largest glacier of Himalaya after Siachen, is one of the extreme high altitude habitats of Himalaya. This glacier is 30 kilometers long with area (∼144 km^2^) and 0.5–2.5 km wide at height from 3000 to 7000 meters above sea level [12]. The permanent cold environment and scarcity of available soil nutrients at Gangotri make it the most stressed ecosystem of WIH. Previously, very few studies documented the bacterial diversity and soil chemical properties in Gangotri glacier ice and soil ecosystems [13]. Being one of the most high altitude ecosystems of WIH, Gangotri represent the most suitable soil system to study the effect of altitudinal variation on bacterial diversity. Thus, comparison of bacterial diversity of Gangotri soil to other low altitude soil system of WIH will provide the better insight of altitudinal variation on soil physiochemical properties and bacterial diversity. Moreover, Gangotri glacier is most affected by the consequences of global warming. Over the last 25 years of the 20th century it has retreated more than 850 meters [14]. Therefore, proper documentation of microbial diversity in soil ecosystem near Gangotri glacier will facilitate the studies of global warming induced changes in microbial community structure in future.

Culturable studies of bacterial diversity in WIH were exclusively based on the routine culturable techniques which are biased to only rapidly growing bacteria those grow only in nutrient rich medium. Thus, majority of unculturable bacteria are not trapped with such techniques [15]. Various attempts were made earlier to increase the isolation spectrum of bacteria from the environmental samples [16]. Previously Kaeberlein *et al*. 2002 [15] used the diffusion chamber based isolation strategy followed by *in situ* incubation for the isolation of novel microorganisms. However, no such attempts were ever made to cultivate bacterial diversity from WIH. Therefore, isolation of microorganisms from Gangotri in their natural soil environment through *in situ* incubation could increase the culturable bacterial diversity. Moreover, psychrophilic bacteria from Himalaya are known for their potential plant growth promotion [17, 18]. Nitrogen fixation in this harsh environment is of great interest because of the occurrence of high energy intensive nitrogen fixation process under cold stress in nutrients poor soil [10]. Comparative study of culturable psychrophilic diazotrophs in different altitude will emphasize the effect of altitude on culturable diazotrophic diversity. Therefore, besides revealing basic molecular mechanisms, psychrophilic diazotrophs from Gangotri could be the potential candidates for the bioinoculant development in the cold climatic agriculture.

Thus keeping above points in mind, soil chemical properties and bacterial diversity of high altitude Gangotri soil were studied and compared with previously studied Kandakhal soil to understand the effect of altitude on soil physiochemical properties and bacterial diversity [19]. Moreover, to assess the effect of altitude on psychrophilic diazotrophs, culturable diazotrophic diversity of both soils were compared. Further, indigenous diffusion chambers were constructed for the bacterial isolation from Gangotri soil as they were never used for WIH soil ecosystems.

## Materials and methods

### Soil sampling

Soil samples were collected from high altitude soil ecosystem of Gangotri (30.9947° N 78.9398° E). Upper 5 cm soil was taken randomly from different places with soil augur, mixed properly and kept in sterile polyethylene bags in cool packed box. Immediately after the transportation, one part of this sample was placed at 4°C for culturable study and other part was kept in -20°C for molecular studies. Some of the physiological parameters of the sampling sites were recorded on the spot and others were collected from the metrological survey. Sampling sites are depicted in **S1 Fig**. Sampling site did not involve endangered or protected species and no specific permissions were required to collect soil samples from this location.

### Soil chemical analysis

Soil samples were analyzed for different soil physiochemical parameters, mainly soil texture, pH, total organic carbon (TOC), total Kjeldhal nitrogen (TKN), total phosphorus (P), nitrates (NO_3_^-^), ammonia (NH_3_), sulphates (SO_4_^-2^) and other trace elements. Soil analysis was out sourced from the Accurate Analytical Laboratory; Pune (ISO 9001–2000; Certified by TU¨ V, Germany).

### Soil DNA extraction

Total soil DNA was extracted from 0.25 g soil sample using Powersoil DNA isolation kit (Mobio Lab. Inc., USA), according to the manufacturer’s instructions. DNA was quantified spectrophotometrically at 260 nm and stored in TE buffer (10 mM Tris, 1 mM EDTA, pH 8.0) at −80°C till further use [18].

### Real-time quantification and DGGE analysis of 16S rDNA and *nif*H genes

Copy number of 16S rDNA and *nif*H genes from collected soil samples were quantified using iCycler iQ5 Multicolor (Bio-Rad Lab, Hercules, USA) real-time polymerase chain reaction (qPCR) machine as described previously [17, 20]. DGGE was performed on a Dcode system (Bio-Rad Lab, Hercules, CA, USA). PCR for 16S rDNA and *nif*H DGGE analysis was performed as per earlier studies and PCR products were separated on 8% (w/v) acrylamide–bisacrylamide gel with a 40–70% denaturing gradient of urea and formamide at 60°C and 90V in 1X TAE for 16 hours [20, 21]. The gels were stained for 30 min in ethidium bromide in 1X TAE (Invitrogen, Paisley, UK) and visualized with a Gel Documentation system (Bio-Rad Lab, Hercules, CA, USA).

### Metagenomic sequencing

Metagenomic sequencing was outsourced at Sandor Proteomics Pvt. Hyderabad, India. Sequencing was performed with two technical replicates. In this analysis, V3 region of the 16S rRNA gene was amplified using primer pair (341F-5′CCTACGGGAGGCAGCAG3′; 518R- 5′ATTACCGCGGCTGCTGG3′). Amplicons were purified and paired-end sequenced on an Illumina Hi-Seq platform. Singletons were removed as it might be a consequence of sequencing errors and can result in spurious OTUs. All the pre-processed reads were used to identify the OTUs using QIIME software package for constructing a representative sequence for each OTU. The representative sequence was finally aligned against Greengenes core set of sequences using PyNAST program. Representative sequence for each OTU was classified using RDP classifier and Greengenes OTU database and the sequences of those not classified were categorized as unknown. NGS data has been deposited to the NCBI Sequence Read Archive (SRA) with accession number SRR8208864.

### Indigenous diffusion chamber and bacterial isolation

**Fig 1:**
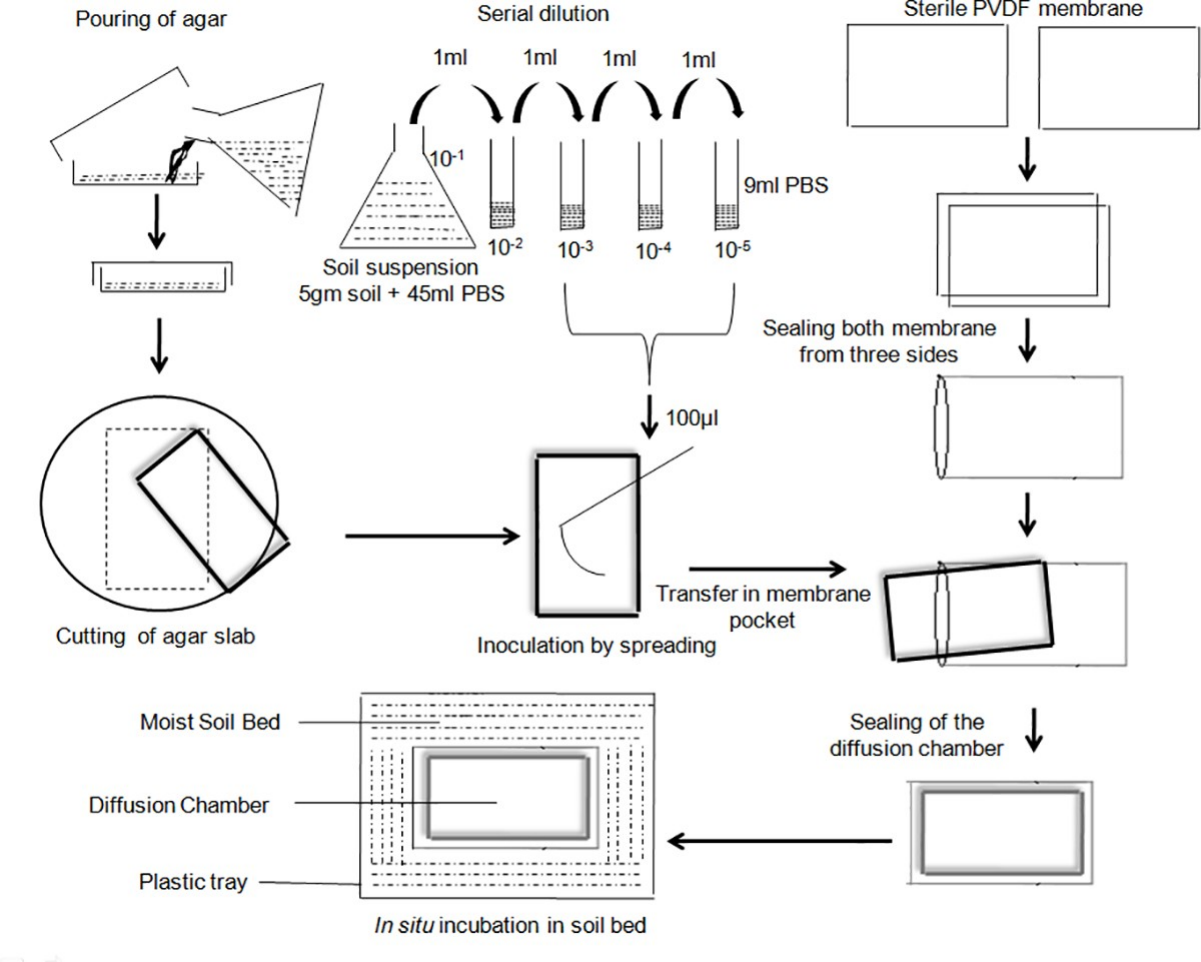
Schematic diagram of the construction, inoculation and incubation of diffusion chamber. Construction of diffusion chamber and inoculation was performed in aseptic conditions under the laminar air flow.

A diffusion chamber was made with the PVDF membrane. For its fabrication two rectangular PVDF membrane pieces were cut (14 × 7 cm) and sterilized through autoclaving. Under aseptic conditions a membrane pocket was made and sealed from the three sides leaving one side open for putting the inoculated agar slab. For making the agar slab, molten agar was poured in sterile Petri plate and cut to the appropriate size with scalpel blade so that it can fit into the pocket. A 100 μL of 10^−3^ dilution of sample was spread on both sides of the slab and placed under the pocket. After putting inoculated agar slab into the pocket, remaining side was sealed and diffusion chamber was kept in the moist soil bed prepared from the soil taken from the original sample site. Diffusion chamber was repeatedly turned to improved diffusion of gases. Several such diffusion chambers were made and incubated in native soil bed for different time intervals. Steps involved in the construction, inoculation and incubation of the diffusion chamber are depicted in **Fig 1.**

### Isolation and characterization of psychrophilic diazotrophs

Soil sample was serially diluted and 100 μL of each 10^−3^ and 10^−4^ dilution was spread on solid nitrogen deficient Burk’s medium and incubated at 2°C for 48 hours. Individual colonies were picked and repeatedly streaked on Burk’s medium and again incubated at 2°C and finally preserved in glycerol stock at -80°C. All the selected isolates were screened for the presence of *nif*H gene through PCR [17]. Thereafter, growth profile with respect to temperature was studied for *nif*H positive isolates and only those isolates having their optimum temperature in psychrophilic range were selected. These selected putative psychrophilic diazotrophs were sequenced and identified at Microbial Culture Collection (MCC) NCCS, Pune, India.

## Results

### Physico-metrological parameters of sampling site

Sampling site at Gangotri had the coordinates 30.9947° N, 78.9398° E and was situated at the altitude of 3,415 meters. Comparative physico-metrological parameters of both the sampling site are given in the **Table 1.** Annual mean maximum and minimum temperature was 11.1 ±0.7°C and **-**2.3 ±0.4°C, respectively. However soil temperature at the time of sampling was 4°C and was found maximum 10°C and minimum **-**2°C for the sampling day. Kandakhal sampling site had the coordinates 29.8800° N and 78.5710° E with altitude 1532 meters. Annual mean maximum and minimum temperature was 25.1 ±0.7°C and 8.3 ±0.5°C, respectively. However soil temperature at the time of sampling in Kandakhal was 12°C and was found maximum 18°C and minimum 10°C for the sampling day. Mean annual snowfall in Gangotri was 257.5 ± 81.6 cm for sampling year and no snowfall was recorded in Kandakhal.

**Table 1:** Comparative physico-metrological parameters of Gangotri and Kandakhal sampling site.

S. No.	Physico-metrological parameter	Gangotri	Kandakhal
1	Sampling time	Late November	Late November
2	Latitude	30.9947° N	29.8800° N
3	Longitude	78.9398° E	78.5710° E
4	Altitude	3,415	1532
5	Soil temperature at the time of sampling	4 ± 05°C	12±1°C
6	Average annual temperature	Max. 11.1 ±0.7°C Min. **-**2.3 ±0.4°C	Max. 25.1 ±0.7°C Min. 8.3 ±0.5°C
7	Average precipitation	140 mm	200mm
8	Mean annual snowfall	257.5 ±81.6 cm	N/A

### Soil analysis

**Fig 2:**
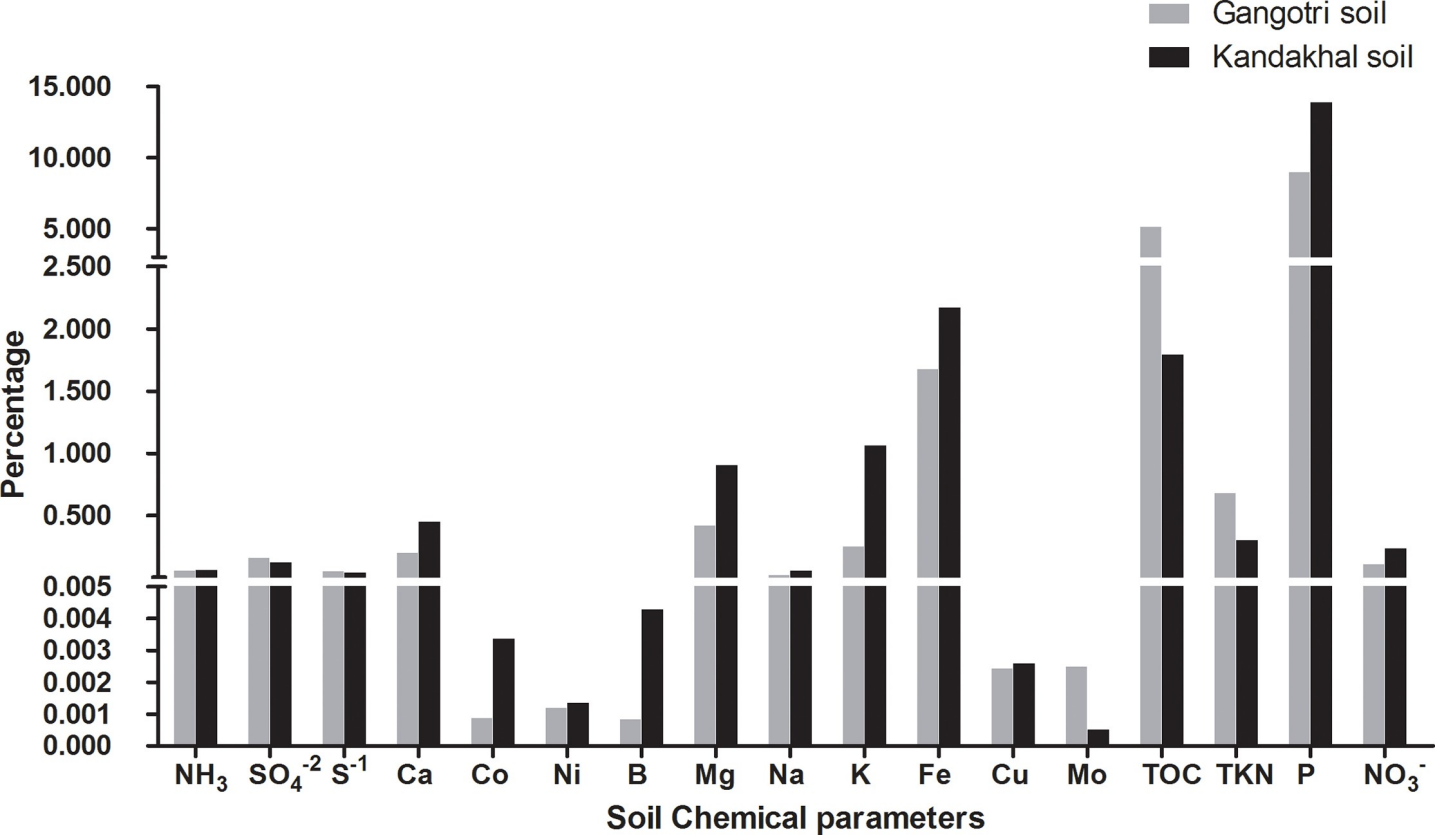
Comparative soil chemical properties of Gangotri and Kandakhal soil.

Soil analysis revealed that the chemical properties of soil vary significantly with the altitude as all the soil chemical parameters were found different in both soils **(Fig 2)**. Soil texture was found coarse in both Gangotri and Kandakhal soil. Soil pH in Gangotri soil was 8.1 which was comparatively higher than Kandakhal where it was 7.2. Total organic carbon (TOC) for Gangotri and Kandakhal soil was 5.1006% and 1.7915%, respectively, whereas, Total Kjeldhal Nitrogen (TKN) was 0.6803% and 0.3033%, respectively. Soil nitrate in Gangotri and Kandakhal soil was 0.1107% and 0.2376%, respectively, while soil ammonium was 0.0538% and 0.0583%, respectively. Total Phosphorus in Gangotri and Kandakhal soil was 8.9387% and 13.8865%, respectively. Further, sulphates (SO_4_^- 2^) in Gangotri soil was 0.1591%, while in Kandakhal soil it was 0.1217%. Soil calcium, cobalt, nickel, boron, magnesium, sodium, potassium, iron and copper were significantly (P<0.005) higher in Kandakhal soil **(S1 Table)**. However, molybdenum was comparatively five times higher in Gangotri soil (0.0025%) as compared to the Kandakhal soil (0.0005%). All the values of these macro-micro nutrients in both soils are given in the **S1 Table**.

### Real-time quantification and DGGE analysis of 16S rDNA and *nif*H

**Fig 3:**
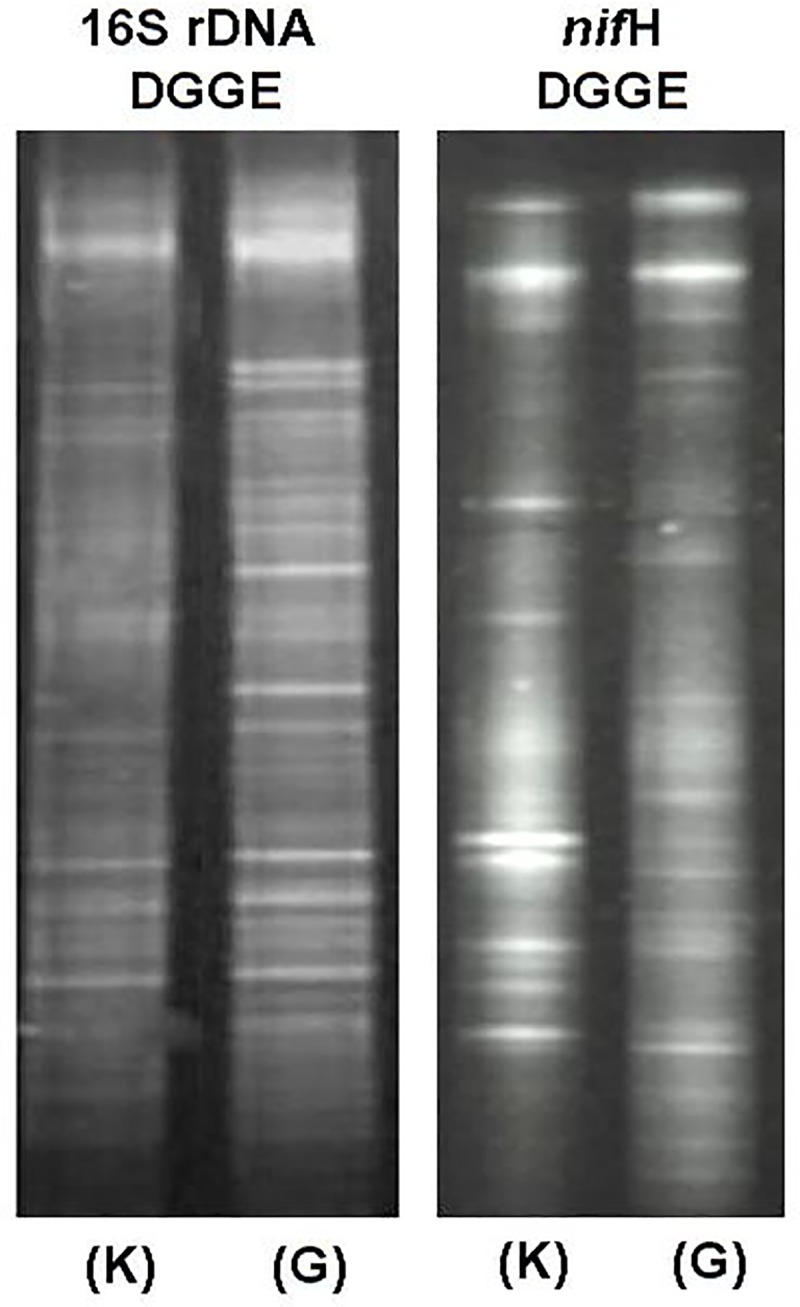
Comparative 16S rDNA (a) and *nif*H (b) DGGE profile in Gangotri and Kandakhal soil.

Real time quantification of 16 S rDNA and *nif*H in Gangotri soil revealed that the copy numbers of 16S rDNA and *nif*H genes were 9.6×10^9^ and 1.1 ×10^5^, respectively. However in Kandakhal soil this was 4.63 × 10^10^and 8.7×10^5^, respectively. In comparison to Kandakhal soil, Gangotri soil had significantly low copy number of 16S rDNA and *nif*H genes. Further, 16S rDNA and *nif*H DGGE profile in both soil showed the different banding pattern which indicates the difference in bacterial and diazotrophic diversity **(Fig 3)**. Comparison of 16S rDNA and *nif* H DGGE profile for both soils revealed the comparatively large OTU in Gangotri, suggesting the rich bacterial and diazotrophic diversity in Gangotri soil despite the low copy number of 16S rDNA and *nif*H genes.

### Bacterial diversity analysis of Gangotri soil

**Fig 4:**
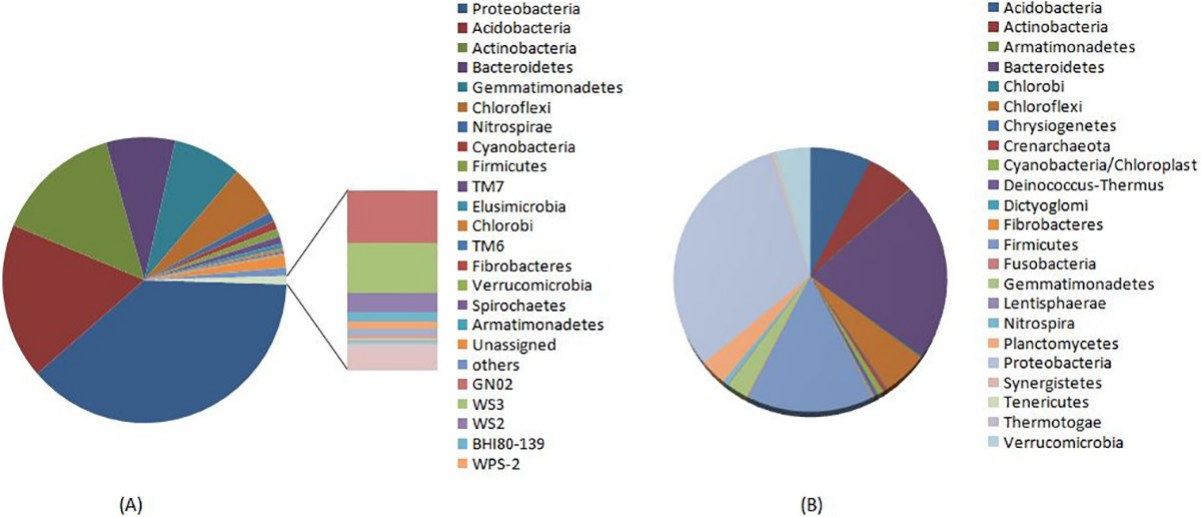
Dominant bacterial phyla in Gangotri (A) and Kandkhal soil (B).

**Fig 5:**
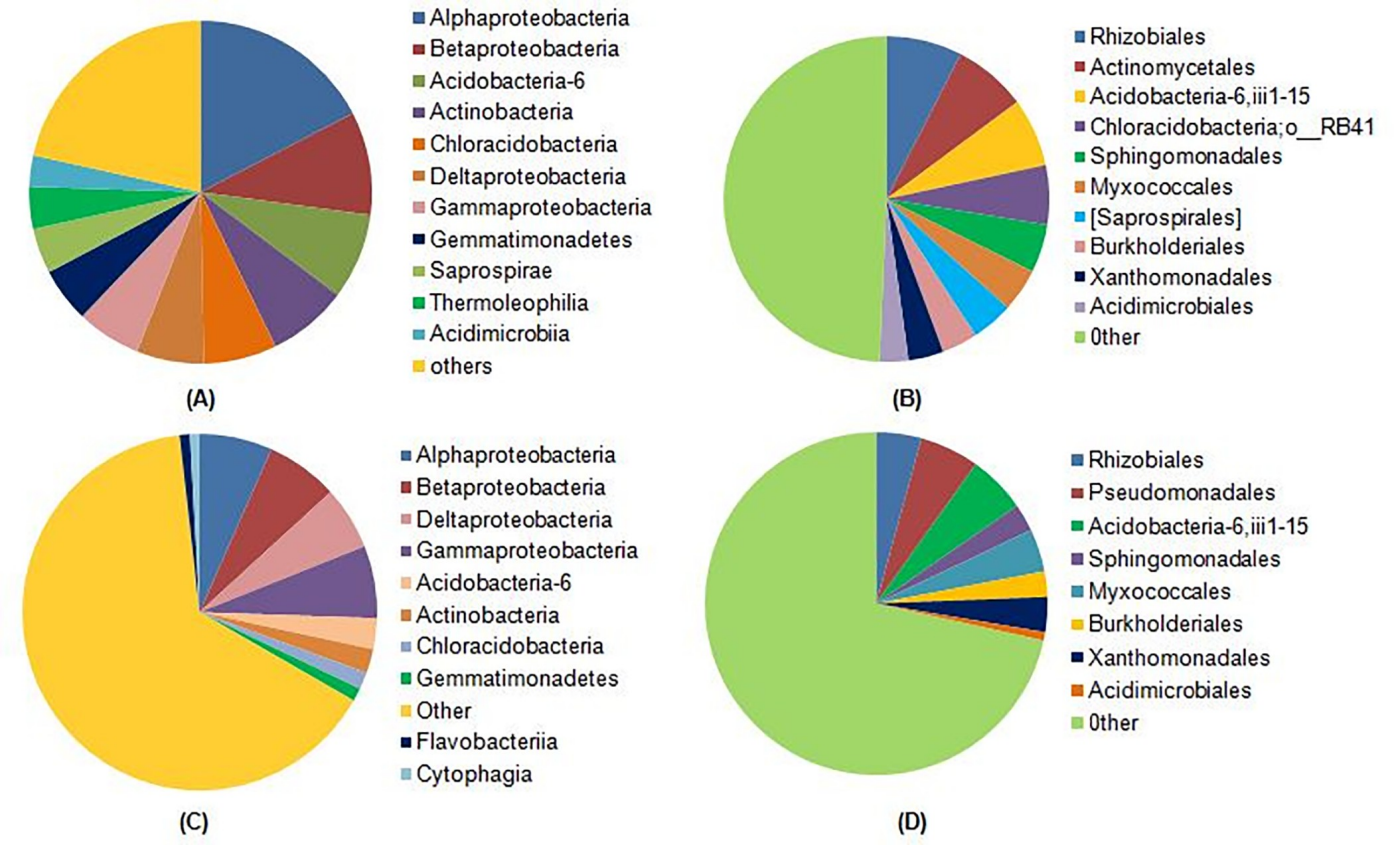
Comparative representation of bacterial taxa in Gangotri and Kandakhal soil. Where, (A) and (B) represents the relative percentage of bacterial class and order in Gangotri soil and (C) (D) represent dominant classes and order in Kandakhal soil.

Metagenomic sequencing of two replicates of Gangotri soil DNA was performed through Illumina Hi-Seq platform. All the sequencing parameters are given in supplementary material **(S2 Table)**. Analysis of metagenomic data revealed 115313 OTU in Gangotri soil. Shannon index (H’), Chao index, Simpson index, Equitability and PD whole tree were found 12.680, 115313, 0.998, 0.754 and 2464.99, respectively. The Shannon’s diversity index considers both richness and evenness while Chao values represent the only richness [22]. Moreover, Gangotri soil harbored diverse lineages of bacterial phyla. A total of 31 bacterial phyla were observed where, *Proteobacteria* (38.49%), *Acidobacteria* (17.88%) *Actinobacteria* (14.48%), *Bacteroidetes* (7.89%), *Gemmatimonadetes* (7.87%), *Chloroflexi*, (5.94%), *Nitrospirae* (1.08%),*Cyanobacteria* (0.93%), *Firmicutes* (0.9%), *TM7* (0.84%), and *Elusimicrobia* (0.55%) were ten dominant bacterial phyla **(Fig 4)**. *Alphaproteobacteria* (16.88%) was the most abundant bacterial class followed by *Betaproteobacteria* (9.44%), *Acidobacteria-6* (7.86%) and *Actinobacteria* (7.33%) **(Fig 5)**. Further analysis revealed that *Rhizobiales* (7.55%), *Actinomycetales* (7.29%), *Acidobacteria-6*,iii1-15 (6.81%), *Chloracidobacteria;o__RB41* (5.89%) and *Sphingomonadales* (4.78%) were dominant orders **(Fig 5)** while, *Sphingomonadaceae* (4.52%), *Hyphomicrobiaceae* (3.79%) and *Chitinophagaceae* (3.42%) were dominant family. Taxonomic analysis showed that at phylum, class, order, family and genus level large number of reads were not identified. Presence of these large numbers of unidentified OTU suggests that the Gangotri soil had unique bacteria diversity.

### Diffusion chamber and culturable diversity

Diverse bacterial species were obtained through diffusion chamber based culture-dependent strategy. Molecular identification of 14 isolates selected on the basis of different colony characteristic revealed them as *Paenarthrobacter nirogunajacolicus*, *Dyadobacter endophyticus*, *Arthrobacter pascens*, *Paenarthrobacter siccitolerans*, *Pseudomonas mandelii*, *Acidovorax facilis*, *Pantoea gaviniae*, *Pseudomonas baetica*, *Pseudomonas frederiksbergensis and Arthrobacter equi* **(Table 2)**. Some of these isolates had never been documented from the WIH and are considered to be unculturable. These results suggest that this indigenous diffusion chamber could facilitate the isolation of novel/unique bacterial species.

**Table 2:** Identification of bacterial cultures isolated through diffusion chamber.

S.No	Culture ID	Top-Hit taxon	Strain	Similarity (%)
1	UC-1	*Paenarthrobacter nirogunajacolicus*	G2-1(T)	99.86
2	UC-2	*Pseudomonas mandelii*	CIP 105273 (T)	99.59
3	UC-3	*Paenarthrobacter nirogunajacolicus*	G2-1(T)	99.86
4	UC-6	*Arthrobacter pascens*	DSM 20545 (T)	99.37
5	UC-8	*Pseudomonas mandelii*	CIP 105273 (T)	99.86
6	UC-9	*Paenarthrobacter siccitolerans*	G2-1(T)	99.86
7	UC-10	*Dyadobacter endophyticus*	65 (T)	97.6
8	UC-11	*Pseudomonas mandelii*	CIP 105273 (T)	99.59
9	UC-13	*Acidovorax facilis*	CCUG 2113(T)	99.06
10	UC-14	*Pantoea gaviniae*	A18/07(T)	98.31
11	UC-15	*Arthrobacter nitroguajacolicus*	G2-1(T)	99.18
12	UC-16	*Pseudomonas baetica*	a390(T)	99.59
13	UC-17	*Pseudomonas frederiksbergensis*	JAJ28(T)	99.46
14	UC-18	*Arthrobacter equi*	IMMIB L-1606(T)	98.76

### Isolation of culturable psychrophilic diazotrophs

Bacterial isolation from Gangotri soil at 2°C in nitrogen deficient medium for 48 hours gave 30 bacteria isolate on the basis of differential colony characteristic. Further, only 25 isolates gave positive result in PCR based *nif*H amplification. Growth profiling of these 25 diazotrophs with respect to temperature suggested that only six have temperature profile in the psychrophilic temperature range **(S2 Fig)**. All these six bacterial isolates had optimum growth temperature of 10°C and barely grow above 25°C. Molecular characterization revealed these psychrophilic diazotrophs as *Pseudomonas helmanticensis*, *Arthrobacter humicola*, *Brevibacillus invocatus and Pseudomonas mandelii* etc **(Table 3)**. Further, no psychrophilic culturable diazotrophic bacteria were isolated from Kandakhal soil in nitrogen deficient medium after prolong incubation at 2°C.

**Table 3:** Molecular characterization of the cold adapted diazotrophs isolated from Gangotri.

S.NO	Sample ID	Top-Hit Taxon	Top-Hit Strain	Similarity (%)
**1**	B-3	*Pseudomonas helmanticensis*	OHA11(T)	99.51
**2**	B-8	*Arthrobacter humicola*	KV-653(T)	98.67
**3**	B-25	*Brevibacillus invocatus*	NCIMB 13772(T)	99.93
**4**	AB-03	*Pseudomonas mandelii*	CIP 105273(T)	99.52
**5**	AB-04	*Pseudomonas helmanticensis*	OHA11(T)	99.31
**6**	AB-16	*Pseudomonas mandelii*	CIP 105273(T)	99.73

## Discussion

This study emphasized the effect of high altitude and associated factors on soil physiochemical properties and bacterial diversity. All the studied soil physiochemical parameters were comparatively different for both soils **(Fig 2)**. Soil organic matter (SOM) is the important factor determining the microbial community structure in soil. In comparison to the Kandakhal soil, TOC was found significantly (P<0.05) higher in Gangotri soil. TOC content in Gangotri and Kandakhal soil was found 5.1006% and 1.7915%, respectively which was three times higher in Gangotri soil. Generally soil with SOM contents <0.5% is considered poor and >2.0% is desirable for agriculture [23]. Further analysis revealed that SOM was negatively correlated with temperature and positively correlated with altitude. Low temperature is reported to decrease the microbial and enzymatic activity in high altitude soil, thus rendering the SOM unaffected by microbial decomposition [24]. Therefore low temperature at high altitude is the major factor determining the high SOM at high altitude.

Total phosphorus (P) was found low, in Gangotri soil than Kandakhal. Further, less acidic pH of Gangotri soil would also reduce the bioavailability of P to the living system. Soil ammonium content was observed almost same in both the soils but soil nitrate and TKN were found two fold higher in Gangotri soil. Levels of ammonium nitrogen in soil vary from 2–10 ppm, but under cold conditions it was reported above 10 ppm [25]. TKN in Gangotri and Kandakhal soil was found 0.6803% and 0.3033%, respectively. Total nitrogen in agriculture soil is generally reported to vary from 0.10% to 0.15%, thus both the soils were rich in total nitrogen. Increased soil temperature is reported as a primary environmental factor that decrease the N mineralization processes thus influencing the bioavailability of soil nitrogen [26]. Nitrogen associated with SOM is not readily mineralized, thus comparatively high total N content of the soil at high altitude could be the result of high SOM. Though, the soil TOC, N and S content was found high in Gangotri soil, their bioavailability to the living system is major factor. Most of the N, P and S remain bound to the SOM, which is not degraded sufficiently under low temperature. Previously, nitrogen mineralization rate, SOM degradation rate and soil P content were found to decrease with temperature [27, 28]. Therefore, low nutrient status of high altitude soil is the result of low temperature induced decrease in mineralization and decomposition.

Further, mineral nutrients analysis revealed that calcium, cobalt, nickel, boron, magnesium, sodium, potassium, iron and copper were found decreased at high altitude soil. Similar trend in micronutrient dynamics with respect to altitudinal variation was found, previously [5]. However, Mo was found comparatively very high in Gangotri soil. Mo is usually present in soil in a concentration of 0.25–5 ppm [29]. High pH could be the possible reason for this increased Mo in Gangotri soil as pH is positively correlated with Mo concentration in soil. Thus Gangotri soil is alkaline, rich in SOM and TKN and poor in mineral nutrients. Therefore, this study showed the negative impact of increasing altitude on soil nutrient status which in turn influences microbial diversity.

Quantitative study of total bacteria and diazotrophs through q-PCR showed that bacterial and diazotrophic counts were significantly (P < 0.05) influenced by altitudinal differences. At Gangotri soil, high altitude and associated stresses decrease the total bacterial and diazotrophic count. Low available carbon and other soil nutrients at high altitude could negatively affect the growth of heterotrophic bacteria thus reducing the total bacterial count (TBC). On the other hand, low available nitrogen could provide selective advantages to the diazotrophs thus enhancing the diazotrophic count to the level which could sustain with other available nutrient resources [30]. Further, high soil nitrate under decreased N mineralization conditions indicates that the high nitrate concentration at high altitude soil could be the functional attribute of the diazotrophs which is further supported by the rich diazotrophic count in Gangotri soil.

Moreover, unculturable study of Gangotri soil concludes that this soil is rich in bacterial diversity as evident by the appearance of large number of different bacterial taxa. Further, presence of many taxonomically unclassified sequences indicates the presence of novel bacterial diversity. Moreover, Chao1 index, Shannon’s diversity index, Simpson index, Equitability and PD whole tree was significantly higher for Gangotri soil than Kandkhal soil. Comparison of metagenomic data of these two soils revealed the dominance of *Proteobacteria* in both soils with comparatively high relative abundance in Gangotri soil than Kandakhal. Besides, *Proteobacteria*, *Acidobacteria* and *Actinobacteria* had high abundance in Gangotri soil, while *Bacteroidetes* and *Fermicute*s were found higher in Kandakhal. *Firmicutes* was the third most abundant phylum in Kandakhal, but in Gangotri their abundance was very low indicating the negative impact of high altitude on the diversity of gram positive bacteria. High ratio of Gram-negative to Gram positive bacteria in Gangotri soil is due to the selective increase in proteobacterial diversity and decrease in the diversity of *Firmicutes*. Thus altitude has profound effect on the ratio of Gram-negative/Gram positive bacteria in the high altitude ecosystems. Many other bacterial phyla, *Bacteroidetes*, *Gemmatimonadetes*, *Nitrospirae*, *Verrucomicrobia*, *Armatimonadetes*, *Cyanobacteria*, *Planctomycetes*, and *Chloroflexi* were present in both soils. The abundance of *Cytophaga*, *Flavobacterium and Bacteroides* (CFB) were found positively correlated with high altitude. Presence of these phyla in cold deserts have also been reported in many studies [31–33].

Community composition of bacteria within above mentioned phyla was significantly different in both soils. In Gangotri, *Alphaproteobacteria* (16.88%) was the most abundant bacterial class of *Proteobacteria* followed by *Betaproteobacteria* (9.44%), *Deltaproteobacteria* (6.17%) and *Gammaproteobacteria* (5.9%). However, in Kandakhal soil all the above mention classes of *Proteobacteria* were uniformly distributed each having 8% abundance. Distribution of proteobacterial class in cold deserts is sensitive to seasonal variation and show dominance of *Betaproteobacteria* in summer, while *Alphaproteobacteria* shows equal abundance throughout the all seasons. Though, *Betaproteobacteria* is reported the most abundant proteobacterial class in high altitude, but little is known about the distribution of *Proteobacteria* in glacier ecosystem [34]. Previous studies of the permafrost glaciers revealed the *Alphaproteobacteria* as a dominant *Proteobacteria* in glacier soil. Thus glacier conditions at Gangotri could be the possible factor determining the dominance of *Alphaproteobacteria* in Gangotri soil.

Incubation in indigenous diffusion chamber resulted in the isolation of bacteria which were least characterized and supposed to be putative novel. This was the first time when culturable bacteria from high altitude cold desert soil were isolated through diffusion chamber based strategy. All the bacteria isolated through this technique were identified as *Paenarthrobacter nirogunajacolicus*, *Dyadobacter endophyticus*, *Arthrobacter pascens*, *Paenarthrobacter siccitolerans*, *Pseudomonas mandelii*, *Acidovorax facilis*, *Pantoea gaviniae*, *Pseudomonas baetica*, *Pseudomonas frederiksbergensis and Arthrobacter equi*. Comparison of these culturable isolates to the unculturable study revealed that these isolates belong to the phylum *Proteobacteria*, *Actinobacteria* and *Bacteroidetes* which were the most dominant bacterial phylum in Gangotri soil. Most of these isolates had never been isolated from the Gangotri and other regions of WIH. Therefore, these results indicate the effectiveness of “Indigenous Diffusion Chamber” for the isolation of rare bacterial species. Moreover, repeated full length sequencing and phylogenetic analysis of one isolate showed that this belongs to the genus *Dyadobacter*, showing highest similarity of 97.6% with *Dyadobacter endophyticus* 65 (T). Previous literature have shown that the *Dyadobacter endophyticus* 65 (T) is the entophyte of the maize plant, however no such cropping was done at high altitude Gangotri [35]. Thus this strain could be the putative novel and is being characterized further.

Psychrophilic bacterial population has been reported to be increased with altitude [36]. Considering the high altitude and permanent cold stress, psychrophilic nitrogen fixing bacteria from Gangotri soil were isolated. Molecular characterization of selected psychrophilic diazotrophs identified them as *Pseudomonas helmanticensis*, *Arthrobacter humicola*, *Brevibacillus invocatus and Pseudomonas mandelii*. To the best of our knowledge these isolates has been reported as cold adapted but not with nitrogen fixing attribute. Further, nitrogen fixation is an enzymatic process and negatively affected by temperature other than the optimal temperature for nitrogenase [37]. However, growth in nitrogen deficient medium at 2°C revealed that the nitrogen fixation machinery in these bacteria was least affected by very low temperature. Thus, molecular study of these isolates could enhance the present knowledge of the molecular mechanism of cold stressed nitrogen fixation at low temperature.

## Conclusion

This study comprehended that the altitude has confounded effects on the both biotic and abiotic factors of the ecosystem. Comparison of high altitude Gangotri soil with Kandakhal soil revealed that soil chemical properties and microbial community structure both varies significantly with altitudinal gradient. This study concludes that high altitudes soil is alkaline, rich in SOM and TKN and poor in mineral nutrients. Moreover, at high altitude, soil microbial count decreases with the increase in the *Cytophaga*, *Flavobacterium* and *Bacteroides* (CFB), while ratio of Gram-negative/Gram positive bacteria, abundance of proteobacteria and culturable psychrophilic bacteria increases. Unculturable and diffusion chamber based study revealed the presence of unique taxonomically unclassified bacteria in this soil. Further, exploration for psychrophilic diazotrophs revealed the presence of functional psychrophilic diazotrophs in Gangotri soil which have good potential to fix nitrogen even at 2°C. These psychrophilic diazotrophs are potential candidates for low temperature bioinoculants. Therefore, WIH ecosystems are unique for their microbial community structure where altitudinal gradient is major factor determining the soil physiochemical properties and microbial community composition.

## Supporting information

S1 Fig Soil sampling sites in Western Indian Garhwal Himalaya. (TIF)

S2 Fig Temperature growth profile of the selected diazotrophic bacteria isolated at 2°C. (JPG)

S1 Table Comparative soil chemical properties of Gangotri and Kandakhal soil. (DOCX)

S2 Table Sequencing parameters for Gangotri soil DNA. (DOCX)
