# Supplementary figures and images for: Microbial diversity and soil physiochemical characteristic of higher altitude
Source: PLoS One. 2019 Mar 15;14(3):e0213844. doi: 10.1371/journal.pone.0213844 (PMC6419999; doi:10.1371/journal.pone.0213844)

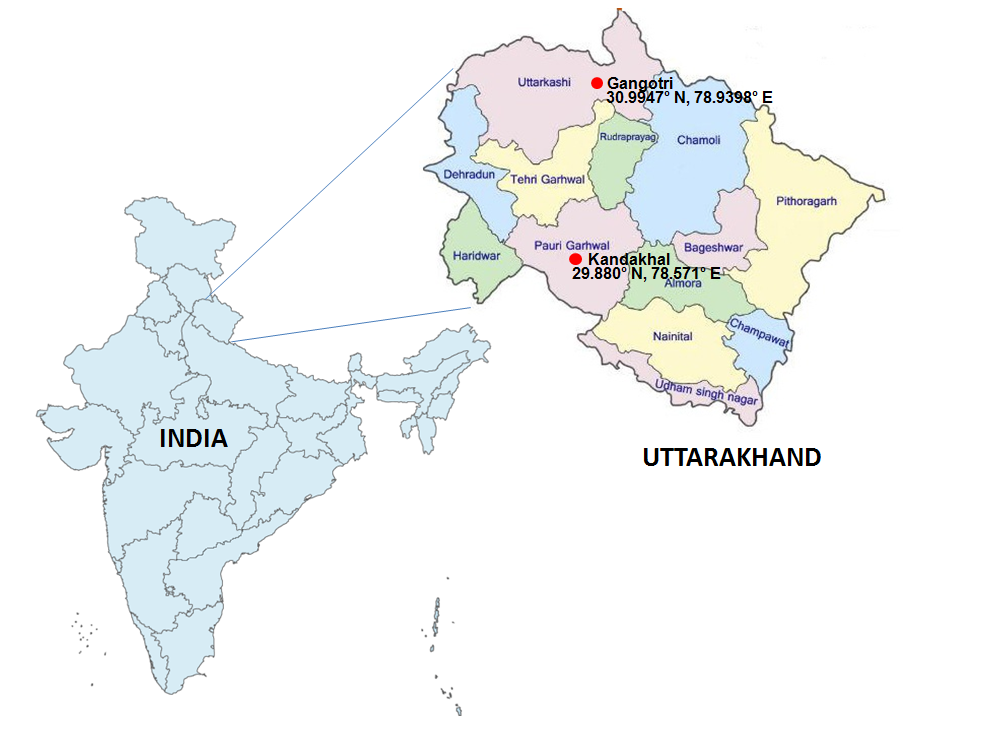

Supplement: S1 Fig — (TIF) [file pone.0213844.s001.tif]

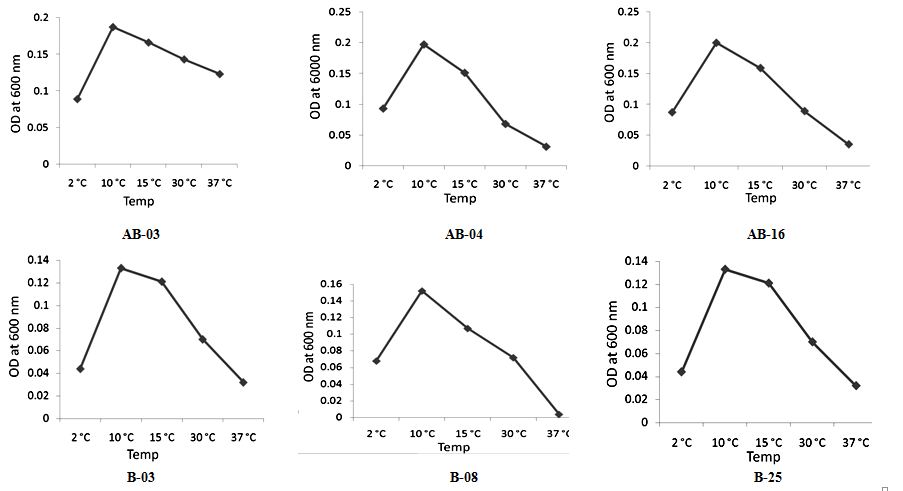

Supplement: S2 Fig — (JPG) [file pone.0213844.s002.JPG]
