# Supplementary material for: Microbial diversity and soil physiochemical characteristic of higher altitude
Source: PLoS One. 2019 Mar 15;14(3):e0213844. doi: 10.1371/journal.pone.0213844 (PMC6419999; doi:10.1371/journal.pone.0213844)
Supplement: S1 Table — (DOCX) [file pone.0213844.s003.docx]

| S.No | Soil test | Unit | Gangotri | Kandakhal |
| --- | --- | --- | --- | --- |
| 1 | Soil pH | **-** | 8.1 | 7.6 |
| 2 | Total organic Carbon ( TOC ) | **%** | 5.1006 | 1.7915 |
| 3 | Total Kjeldhal Nitrogen ( TKN ) | **%** | 0. 6803 | 0.3033 |
| 4 | Total Phosphorus (P ) | **%** | 8.9387 | 13.886 |
| 5 | Nitrates ( NO_3_ ^-^  ) | **%** | 0.1107 | 0.2376 |
| 6 | Ammonia ( NH_3_ ) | **%** | 0.0538 | 0.0583 |
| 7 | Sulphates ( SO_4_ ^- -^ ) | **%** | 0.1591 | 0.1217 |
| 8 | Sulphates (S­^-­^) | **%** | 0.0530 | 0.0405 |
| 9 | Calcium as ( Ca ) | **%** | 0.2012 | 0.4495 |
| 10 | Cobalt (Co) | **%** | 0. 0008 | 0.0033 |
| 11 | Nickel ( Ni ) | **%** | 0.0012 | 0.0013 |
| 12 | Boron (B) | **%** | 0.0008 | 0.0042 |
| 13 | Magnesium (Mg) | **%** | 0.4161 | 0.9037 |
| 14 | Sodium (Na) | **%** | 0.0186 | 0.0572 |
| 15 | Potassium (K) | **%** | 0.2531 | 1.0604 |
| 16 | Iron (Fe) | **%** | 1.6721 | 2.1713 |
| 17 | Copper (Cu) | **%** | 0.0024 | 0.0025 |
| 18 | Molybdenum ( Mo) | **%** | 0.0025 | 0.0005 |

**S1 Table.** Comparative soil chemical properties of Gangotri and Kandakhal soil
