# Supplementary material for: Microbial diversity and soil physiochemical characteristic of higher altitude
Source: PLoS One. 2019 Mar 15;14(3):e0213844. doi: 10.1371/journal.pone.0213844 (PMC6419999; doi:10.1371/journal.pone.0213844)
Supplement: S2 Table — (DOCX) [file pone.0213844.s004.docx]

| **S.No.** | **Sequencing parameters** | **Gangotri soil replicate 1** | **Gangotri soil replicate 2** |
| --- | --- | --- | --- |
| **1** | Total number of bases | 848799750 | 848799750 |
| **2** | Total number of reads | 5658665 | 5658665 |
| **3** | % bases >= Q20 | 96.56 | 91.15 |
| **4** | % bases >= Q30 | 92.67 | 81.69 |
| **5** | Average read length | 150 | 150 |
| **6** | Max read length | 150 | 150 |
| **7** | Min read length | 150 | 150 |
| **8** | Number of base A | 211418924 | 162204896 |
| **9** | Number of base T | 159877575 | 207476306 |
| **10** | Number of base G | 283291094 | 220435216 |
| **11** | Number of base C | 194108151 | 258561263 |
| **12** | Number of base N | 104006 | 122069 |
| **13** | GC content % | 56.24 | 56.43 |

**S2 Table.** Sequencing parameters for Gangotri soil DNA
